# Retraction: Intracrinology and Testosterone Pellet Therapy: An Enzyme-Aware, Symptom-Driven Approach to Hormone Optimization in Aging

**DOI:** 10.7759/cureus.r244

**Published:** 2026-09-10

**Authors:** Rebecca Glaser

**Affiliations:** 1 Surgery, Wright State University Boonshoft School of Medicine, Dayton, USA

This article has been retracted by the Editor-in-Chief. A post-publication review was carried out after the author self-reported several citation errors. The review established that references cited in the article do not support the statements attributed to them, and the conclusions are therefore not reliable. The review identified no data fabrication, falsification, or other research misconduct. The author agrees with the decision to retract.

